# Bone Morphogenetic Protein Type I Receptor Antagonists Decrease Growth and Induce Cell Death of Lung Cancer Cell Lines

**DOI:** 10.1371/journal.pone.0061256

**Published:** 2013-04-12

**Authors:** Elaine Langenfeld, Charles C. Hong, Gandhi Lanke, John Langenfeld

**Affiliations:** 1 Department of Surgery, Division of Thoracic Surgery, UMDNJ-Robert Wood Johnson Medical School, New Brunswick, New Jersey, United States of America; 2 Department of Surgery, UMDNJ-Robert Wood Johnson Medical School, New Brunswick, New Jersey, United States of America; 3 Research Medicine, Veterans Affairs TVHS, Vanderbilt University School of Medicine, Nashville, Tennessee, United States of America; 4 Division of Cardiovascular Medicine, Vanderbilt University School of Medicine, Nashville, Tennessee, United States of America; H. Lee Moffitt Cancer Center & Research Institute, United States of America

## Abstract

Bone morphogenetic proteins (BMPs) are highly conserved morphogens that are essential for normal development. BMP-2 is highly expressed in the majority of non-small cell lung carcinomas (NSCLC) but not in normal lung tissue or benign lung tumors. The effects of the BMP signaling cascade on the growth and survival of cancer cells is poorly understood. We show that BMP signaling is basally active in lung cancer cell lines, which can be effectively inhibited with selective antagonists of the BMP type I receptors. Lung cancer cell lines express alk2, alk3, and alk6 and inhibition of a single BMP receptor was not sufficient to decrease signaling. Inhibition of more than one type I receptor was required to decrease BMP signaling in lung cancer cell lines. BMP receptor antagonists and silencing of BMP type I receptors with siRNA induced cell death, inhibited cell growth, and caused a significant decrease in the expression of inhibitor of differentiation (Id1, Id2, and Id3) family members, which are known to regulate cell growth and survival in many types of cancers. BMP receptor antagonists also decreased clonogenic cell growth. Knockdown of Id3 significantly decreased cell growth and induced cell death of lung cancer cells. H1299 cells stably overexpressing Id3 were resistant to growth suppression and induction of cell death induced by the BMP antagonist DMH2. These studies suggest that BMP signaling promotes cell growth and survival of lung cancer cells, which is mediated through its regulation of Id family members. Selective antagonists of the BMP type I receptors represents a potential means to pharmacologically treat NSCLC and other carcinomas with an activated BMP signaling cascade.

## Introduction

The Bone Morphogenetic Proteins (BMPs) are members of the Transforming Growth Factor superfamily (TGF). BMPs are phytogenetically conserved proteins required for embryonic development from insects to humans. Approximately 20 BMP ligands have been identified and categorized into several subclasses. BMP-2 and BMP-4 share 92% homology and have interchangeable biological activity. BMPs are secreted proteins that signal through transmembrane serine/threonine kinases called type I and type II receptors [1]. The type I receptors are alk1, alk2 (ActR-1), alk3 (BMPR-IA), and alk6 (BMPR-IB) [1]. The type II receptors are BMPR-II and activin type II receptors ActR-II and AcR-IIB [1]. BMP receptors are promiscuous, and can be activated by several BMP ligands [1], [2]. Each BMP ligand is also capable of activating different receptors [1],[2]. Binding of the BMP ligands to the type I receptor leads to phosphorylation by the constitutively active type II receptor. The receptor complex phosphorylates Smad-1/5, which then activates the transcription of downstream target genes [3].

During embryonic development, BMPs regulates cell fate decisions, cell survival, and vasculogenesis [4], [5], [6], [7], processes that are also common in carcinogenesis. In fact, BMP-2 is over-expressed in 98% of NSCLC and other carcinomas [8], [9]. BMP expression inversely correlates with survival [10] and high expression is associated with metastatic spread [11], [12]. BMP-2 enhances tumor angiogenesis [13], [14], [15] and stimulates tumor invasion [8]. Ectopic expression of BMP-2 in A549 lung cancer cells greatly enhanced metastatic growth in a murine model of lung cancer following tail vein injection [16]. Studies using recombinant BMP proteins or knockdown of a single BMP receptor have suggested that BMP signaling in cancer cells does not promote cell growth and may even act as a tumor suppressor (16–19). The effects of inhibiting multiple BMP receptors on cell growth and survival in cancer cells has not been examined. Therefore, the biological significance of a basally active BMP signaling cascade in cancer cells is not known.

During development the inhibitors of DNA binding/differentiation (Id) are direct mediators of BMP signaling. There are 4 Id family members (Id1, Id2, Id3, and Id4). BMP response elements (BRE) on the Id1, Id2, and Id3 promoters are activated by Smad 1/5/8 (20–23). The Id proteins inhibit lineage commitment by binding and sequestering basic HLH transcription factors [17]. Id family members have been implicated in oncogenic transformation in several types of cancers [18] [19], [20]. Id1 has been reported to regulate invasion, proliferation, survival, and the metastatic spread of cancer cells [18], [21], [22]. Id family members are frequently expressed in non-small cell lung carcinomas [23], [24] and over-expression is associated with a shorter disease free survival [25]. These studies suggest that targeting signaling pathways, which regulate the expression of Id family members may have important therapeutic implications. Although recombinant BMP2 proteins induce a transient increase in the expression of Id1 in lung cancer cells [8], the role of the BMP signaling cascade in regulating the basal expression levels of the Id family members in cancer cells has not been elucidated.

The aim of this study was to determine in lung cancer cell lines, which have not been stimulated with a recombinant BMP protein, whether cells have a basally active BMP signaling and determine its effect on cell growth, survival, and expression of Id family members. Selective BMP type I receptor antagonists and siRNA targeting the BMP type I receptors reveals that basally active BMP signaling in lung cancer cell lines is growth promoting and an important regulator of the expression of Id family members. BMP signaling is mediated through more than one type I BMP receptor. DMH2 caused the greatest inhibition of BMP signaling and induced the greatest reduction of cell growth and expression of Id family members.

## Results

### BMP Type I Receptor Antagonists Decrease Smad 1/5/8 signaling

**Figure 1:**
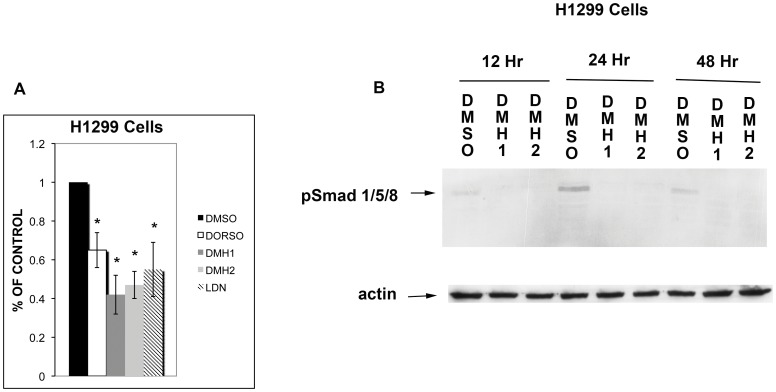
BMP type I receptor antagonists decrease Smad 1/5/8 activity in H1299 cells. (**A**) H1299 cells were transfected with BRE-luciferace reporter. After 48 hours the cells were treated with DMSO, 10 µM Dorsomorphin, or 1 µM DMH1, 1 µM DMH2, or 1 µM LDN for 48 hours. BRE-luciferace activity is reported as the percent of the DMSO control treated cells. Data represents the mean of 3 experiments in triplicate. The mean of the control cells was compared to the mean of the treated cells. * p<0.05. (**B**) Immunoblot analysis for phosphorylated Smad 1/5/8 of H1299 cells treated with 1 µM DMSO, DMH1, or DMH2 for 12, 24, and 48 hours.

Using a BMP-responsive luciferase reporter (BRE-Luc), we examined the effects of the different BMP type receptor antagonists on Smad 1/5/8 activity in H1299 cells. Dorsomorphin, DMH1, DMH2, and LDN all caused a significant decrease in the expression of the BRE-Luc reporter in H1299 cells, indicating a decrease in Smad 1/5/8 activity (figure 1A). Immunoblot analysis revealed that selective BMP type I receptor antagonists decrease phosphorylation of Smad 1/5/8 in H1299 and A549 cells (figure 1B and figure S1). Phosphorylation of Smad 1/5/8 was decreased within 24 hours of treatment and persisted for at least 48 hours thereafter.

### BMP Type I Receptor Antagonists Decrease Expression of Id Family Members

**Figure 2:**
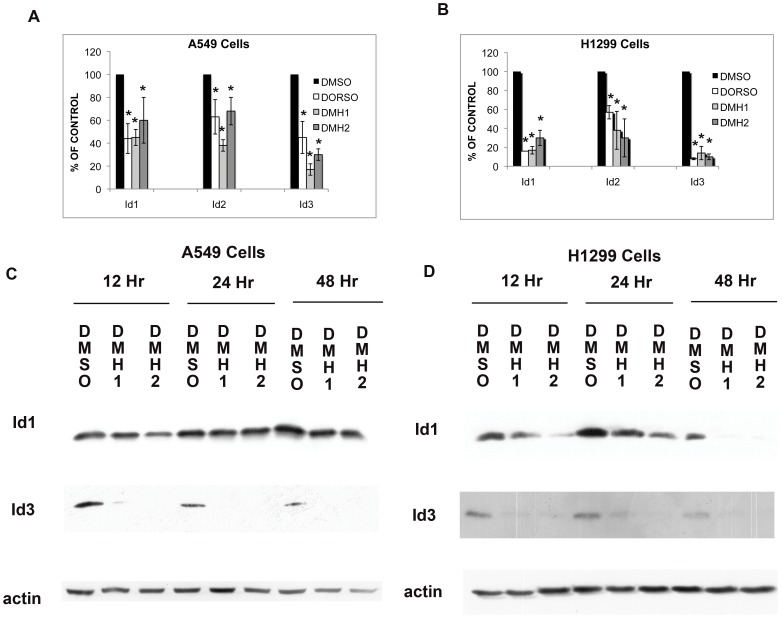
BMP antagonists decrease the expression of Id family members in A549 and H1299 cells. (**A–B**) Quantitative RT-PCR for Id1, Id2, and Id3 on (**A**) A549 and (**B**) H1299 cells treated with DMSO, 10 µM Dorsomorphin, 1 µM DMH1, or 1 µM DMH2 for 48 hours. Data represents the mean of at least 3 experiments performed in duplicate and presented as the percent of control treated cells. The mean of the control cells was compared to the mean of the treated cells. * p<0.05. (**C–D**) Western blot analysis for Id1 and Id3 on (**C**) A549 and (**D**) H1299 cells treated with 1 µM DMSO or 1 µM of selective BMP type I receptor antagonist for 12, 24, and 48 hours. These studies were performed at least 3 times.

Quantitative RT-PCR was used to determine whether the BMP signaling cascade regulates the expression of Id family members in lung cancer cell lines. Dorsomorphin, DMH1, and DMH2 significantly decreased Id1, Id2, and Id3 expression in A549 and H1299 cell lines (figure 2A–B). The BMP antagonists caused a greater reduction of Id family members in the H1299 cells compared to A549 cells. By Western blot analysis, Dorsomorphin, DMH1, DMH2, and LDN caused a decrease in protein levels of Id1 and Id3 in A549 and H1299 cells (figure 2C–D and figure S2). The BMP antagonists decreased the expression of Id family members within 12 to 24 hours that persisted for at least 48 hours. DMH1 and DMH2 caused a greater reduction of Id1 in H1299 cells compared to A549 cells. DMH2 consistently caused a greater reduction of Id1 protein expression than DMH1.

### Multiple BMP Type I Receptors Mediate Signaling

**Figure 3:**
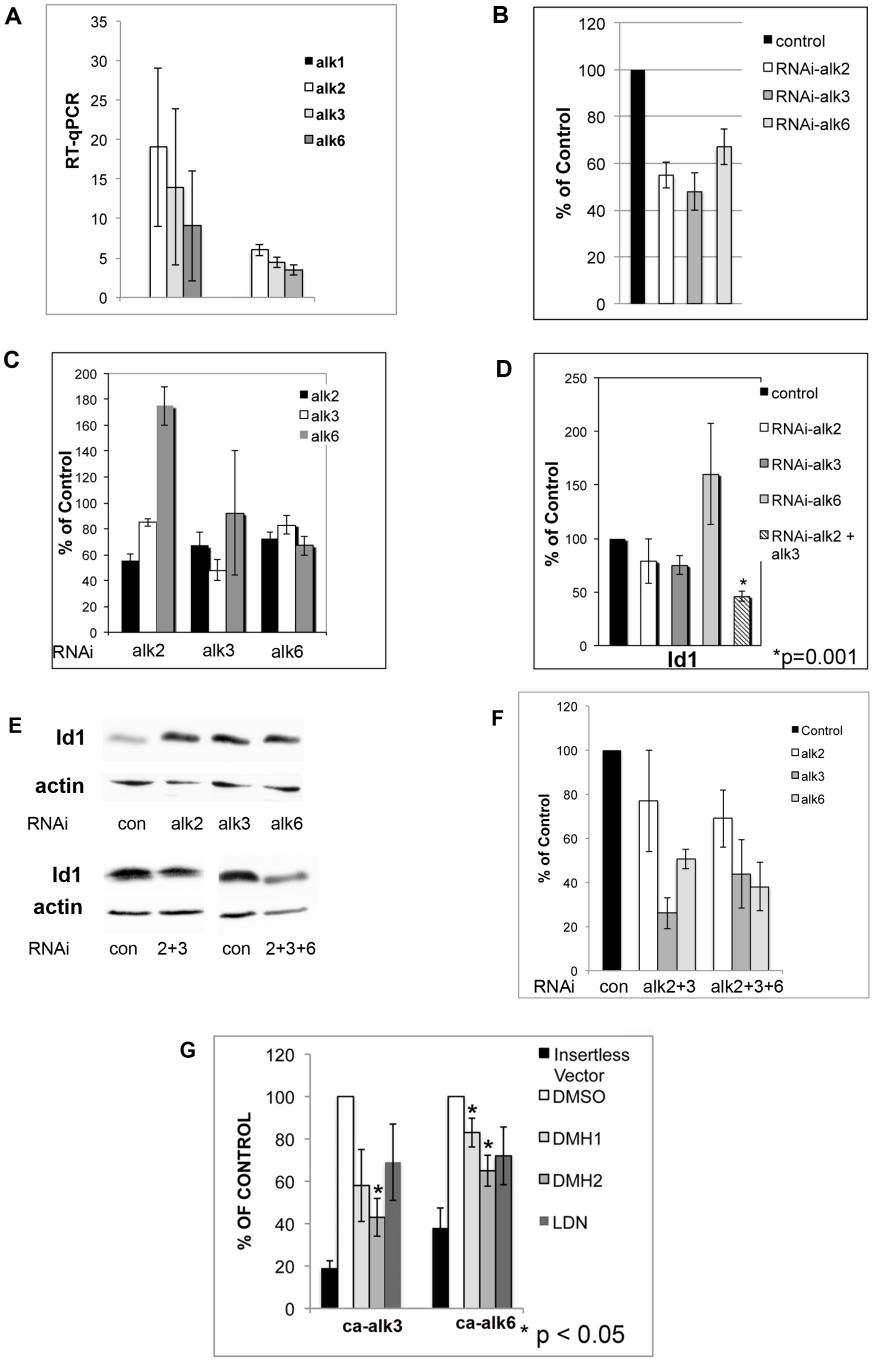
Multiple BMP type I receptors mediate BMP signaling in lung cancer cell lines. (**A**) Quantitative RT-PCR of the A549 and H1299 cells showing expression of alk2, alk3, and alk6 but not alk1 (n = 3). (**B**) H1299 cells were transfected with siRNA targeting each type I BMP receptor and quantitative RT-PCR was performed for that BMP receptor. (**C**) Knockdown of each BMP type I receptor in H1299 cells was performed and quantitative RT-PCR performed for all 3 type I receptors. (**B–C**) Data represents the mean of 2 experiments performed in duplicate. (**D**) Knockdown in H1299 cells of a single BMP type I receptor or both alk2 and alk3. After 48 hours the expression of Id1 was examined by quantitative RT-PCR. Data represents the mean of 3 experiments performed in duplicate. (**E**) Western blot analysis for Id1 in H1299 cells with knockdown of a single type I BMP receptor or combination knockdown of alk2 and alk3, or all 3 BMP type I receptors. Studies were done at least 2 times. Studies show silencing more than one receptor is required to decrease Id1 expression. (**F**) Quantitative RT-PCR of the type I BMP receptors after knockdown of alk2 and alk3, or all 3 BMP type I receptors. Studies done 3 times in duplicate reported as percent of control. (**G**) H1299 cells were co-transfected with insertless vector, constitutively active alk3 (ca-alk3), or constitutively active alk6 (ca-alk6) expression vectors and the BRE-luciferase reporter. Cells were then treated with 1 µM DMSO or 1 µM BMP receptor antagonist for 24 hours and luciferace activity measured. Data demonstrates the mean of at least 3 independent experiments shown as the percent of control.

Next, we assessed whether a specific BMP type I receptor mediates basally active BMP signaling in lung cancer cell lines. By quantitative RT-PCR, the expression of BMP type I receptors was examined. Alk1 was not expressed in either A549 or H1299 cells (figure 3A). As expected, alk1 was expressed in human endothelial cells (data not shown). Alk2, alk3, and alk6 mRNA are expressed in A549 and H1299 cell lines (figure 3A). Using siRNA, the expression of each type I receptor was reduced by approximately 40% or greater (figure 3B). To test specificity of the siRNA, knockdown of each type I receptor was performed and RT-PCR performed for alk2, alk3, and alk6. Knockdown of alk2 caused a significant increase in the expression of alk6 (figure 3C). Knockdown of alk3 and alk6 caused a small decrease in the expression of the other type I receptors (figure 3C). Quantitative RT-PCR showed that silencing of alk2 or alk3 in the H1299 cells caused an approximately 20–25% decrease in the expression of Id1 mRNA (figure 3D), while silencing of alk6 tended to cause an increase in Id1 expression. Silencing both alk2 and alk3 caused a significantly greater decrease in Id1 expression than knockdown of either receptor alone (figure 3D). Western blot analysis showed that knockdown of a single type IA receptor alone did not decrease Id1 expression (figure 3E). Silencing of both alk2 and alk3 or silencing of alk2, alk3, and alk6 did cause a decrease in Id1 protein expression (figure 3E). Quantitative RT-PCR demonstrated that knockdown of multiple receptors led a decrease in all type BMP receptors, which was greater than that seen for single receptor knockdowns (figure 3F).

A second set of siRNA targeting each BMP type I receptor was used to further assess BMP signaling. These siRNA also caused a significant decrease in expression of the targeted receptor in the A549 and H1299 cells (figure S3). Examining the specificity of these siRNA also demonstrated that knockdown of a single receptor caused some downregulation of the other type I BMP receptors (figure S3). These data suggest that there is cross regulation between the different type I BMP receptors. Again, knockdown of a single type I BMP receptor was not sufficient to decrease Id1 expression in either the A549 and H1299 cells (figure S3). To further examine BMP receptor signaling, each receptor was silenced and Smad 1/5/8 activity determined using the BRE-luciferase assay. Silencing only one BMP type I receptor did not cause a decrease in Smad-1/5/8 activity (figure S3). By qRT-PCR, knockdown of all 3 type I BMP receptors in the A549 cells caused a significant reduction in the expression of Id1 (figure S3). The knockdown of all type I BMP receptors (alk2, alk3, and alk6) was confirmed by qRT-PCR (figure S3). Western blot analysis again showed that knockdown of a single receptor in the H1299 cells was not sufficient to decrease the expression of Id1 (figure S3). Consistent with our other set of siRNA, knockdown of alk2+ alk3 or alk2, alk3, and alk6 caused a reduction in protein expression of Id1 (figure S3). These data support that BMP signaling is mediated through more than one type I BMP receptor in lung cancer cell lines.

In the C2C12 mouse myoblast cell line, DMH1 inhibited alk2 and alk3 activity but was reported to have negligible inhibitory effects on alk6 [26]. DMH2 is known to inhibit alk2 and LDN effectively inhibits alk2, alk3, and alk6 [26]. To test receptor selectivity of DMH1, DMH2, and LDN in lung cancer cells, constitutively active alk3 (ca-alk3) or alk6 (ca-alk6) was co-transfected with the BRE-luciferace reporter into H1299 cells (figure 3G). DMH2 caused a greater reduction of alk3 activity than DMH1 and LDN in the H1299 cells. The BMP antagonists caused some inhibition of alk6 but less than that seen for alk3 (figure 3G).

### Inhibition of BMP Type I Receptors Decreases Cell Growth

**Figure 4:**
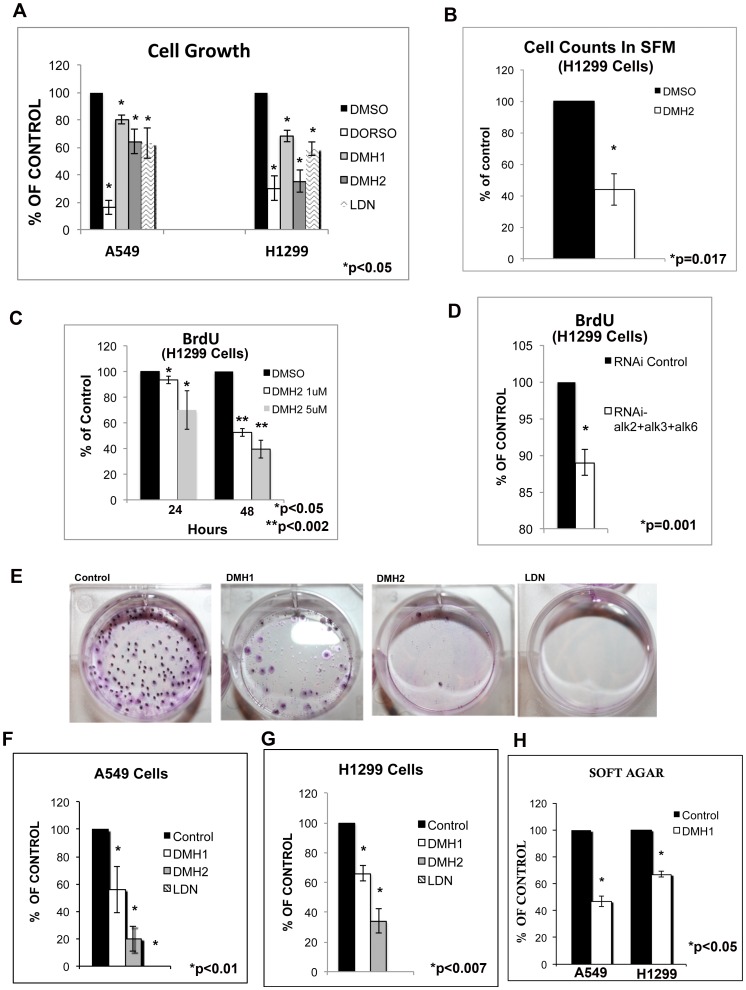
Antagonizing BMP type I receptors decreases cell growth, proliferation, and clonogenicity of lung cancer cell lines. (**A**) A549 and H1299 cells cultured in DMEM 5% FCS were treated with DMSO, 10 µM Dorsomorphin, 1 µM DMH1, 1 µM DMH2, or 1 µM LDN for 7 days and cell counts performed. (**B**) H1299 cells cultured in SFM were treated with 1 µM DMSO or 1 µM DMH2 for 7 days and cell counts performed. (**C**) BrdU incorporation of H1299 cells treated with DMSO or 1 µM or 5 µM DMH2 for 24 and 48 hours. (**D**) BrdU incorporation of H1299 cells transfected with siRNA targeting of all type I BMP receptors or siRNA control. (**C–D**) Data is the mean of 3 experiments in triplicate reported as the percent of control treated cells. (**E–G**) Colony growth of A549 and H1299 cells treated with 1 µM DMSO or 1 µM of selective BMP receptor antagonist. (**E**) Photograph of a representative experiment. (**F–G**) The data shows the mean of at least 3 independent experiments reported as the percent of control. (**G**) DMH1 decreases anchorage independent growth of lung cancer cell lines. A549 and H1299 cells in soft agar were treated with 1 µM DMS0 or 1 µM DMH1 for 2 weeks and the number of colonies counted. The data shown is the mean of 3 independent experiments reported as the percent of control.

Next, the effects of blocking BMP type I receptors on cell growth was examined by performing cell counts. BMP type I receptor antagonists caused a significant reduction in the number of cells after 7 days in both the A549 and H1299 cell lines (figure 4A). The selective BMP receptor antagonists caused a greater reduction in cell growth in the H1299 cells compared to A549 cells. DMH2 caused significantly more growth inhibition than DMH1 in both cell lines (figure 4A). To remove potential BMP ligands in the cell culture medium, the H1299 cells were cultured in serum free medium and treated with DMH2. DMH2 also caused significant inhibition of cell growth of H1299 cells cultured in serum free medium (SFM) (figure 4B), suggesting that BMP signaling may occurs in a self-autonomous manner. Proliferation was examined by determining bromodeoxyuridine (BrdU) incorporation. DMH2 caused a dose dependent decrease in proliferation of the H1299 cells within 24 hours (figure 4C) and a more profound effect was seen at 48 hours (figure 4C). Knockdown of all 3 type I BMP receptors (alk2, alk3, and alk6) in the H1299 cells also caused a significant decrease in BrdU incorporation (figure 4D). Knockdown of alk2, alk3, and alk6 using the second set of siRNA also caused a significant reduction of proliferation in the H1299 cells (figure S3). Quantitative RT-PCR showed that with this second set of siRNA targeting all 3 type I BMP receptors in the H1299 cells caused downregulation of alk2 and alk6 but not alk3 (figure S3). This suggests that inhibition of alk2 and alk6 is sufficient to decrease BMP signaling in H1299 cells.

### BMP Type I Receptor Antagonists Decrease Clonogenic Growth

The effect of BMP receptor antagonists on clonogenic cell growth was examined. DMH1, DMH2, and LDN all caused a significant reduction of clonogenic growth in both the A549 and H1299 cell lines (figure 4E–G). Clonogenic growth was inhibited more by DMH2 and LDN than DMH1 (figure 4E–G). To further assess the effects of DMH1 on clonogenic growth, anchorage independent growth was examined. DMH1 significantly reduced anchorage independent growth in both A549 and H1299 cells (figure 4H). These data show that basally active BMP signaling stimulates proliferation and enhances clonogenic growth of lung cancer cells.

### Inhibition of BMP Type I Receptors Induces Cell Death

**Figure 5:**
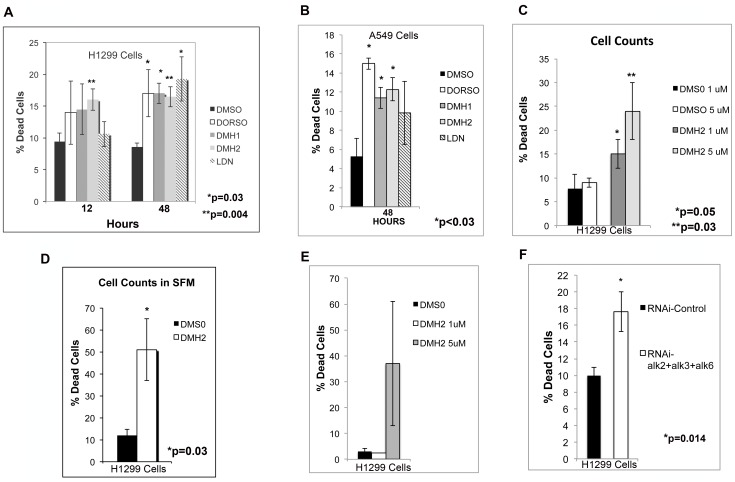
Inhibition of type I BMP receptors in lung cancer cell lines induces cell death. (**A–B**) H1299 and A549 cultured in DMEM 5% FCS were treated with DMSO or a BMP receptor antagonist (10 µM Dorsomorphin or 1 µM of selective antagonist). The percentage of cells that take up ethidium bromide was then determined. The data is reported as the mean of at least 3 independent experiments. (**C**) H1299 cells cultured in DMEM 5% FCS were treated with DMSO, 1 µM and μ5 M of DMH2 for 7 days, stained with Typan Blue, and cell counts performed. Data represents the mean of 4 experiments reported as the percent dead cells. (**D**) H1299 cells cultured in SFM were treated with DMSO or 1 µM of DMH2 for 7 days, stained with Typan Blue, and cell counts performed. Data represents the mean of 5 experiments reported as percent dead cells. (**E**) Cell death was determined using flow cytometry detecting uptake of an amine-reactive fluorescent dye in H1299 cells treated with DMS0 or DMH2 for 4 days. Data represent the mean of 3 independent experiments. (**F**) The H1299 cells were transfected with control siRNA and siRNA targeting alk2, alk3, and alk6. After 2 days the percentage of cells staining for ethidium bromide was determined. The data is reported as the mean of 6 independent experiments.

The effect of BMP signaling on cell survival was examined. Cell death was quantified using an ethidium bromide uptake assay. Ethidium bromide is only taken up by cells that have lost membrane integrity, which occurs when cells are dying by necrosis or apoptosis [27]. BMH2 induced cell death in the H1299 cells within 12 hours, and by 48 hours all of the antagonists caused a significant increase in the percentage of dead cells (figure 5A). Within 48 hours, Dorsomorphin, DMH1, and DMH2 caused a significant increase in cell death in the A549 cells (figure 5B). To further assess cell death, H1299 cells cultured in DMEM 5% FCS were treated with DMH2 for 4 days and the percentage of dead cells determined by Trypan Blue staining. DMH2 caused a significant increase in cell death, which was dose dependent (figure 5C). The percentage of dead cells was even higher in H1299 cells cultured in SFM (figure 5D). An amine-reactive fluorescent dye was used to detect cell death, which also showed that DMH2 induced cell death in the H1299 cells (figure 5E). To further verify that BMP antagonists induce cell death by inhibition of BMP signaling, triple knockdown of alk2, alk3, and alk6 receptors was performed. Knockdown of alk2, alk3, and alk6 receptors caused a significant increase in the percentage of dead cells in comparison to siRNA control treated cells (figure 5F). A second set of siRNA targeting alk2, alk3, and alk6 also caused a significant increase in the percentage of death cells (figure S3). These studies show that antagonizing the activity of the BMP type I receptors induces cell death in lung cancer cells.

### BMP Signaling is Regulated in a Self-autonomous Manner

**Figure 6:**
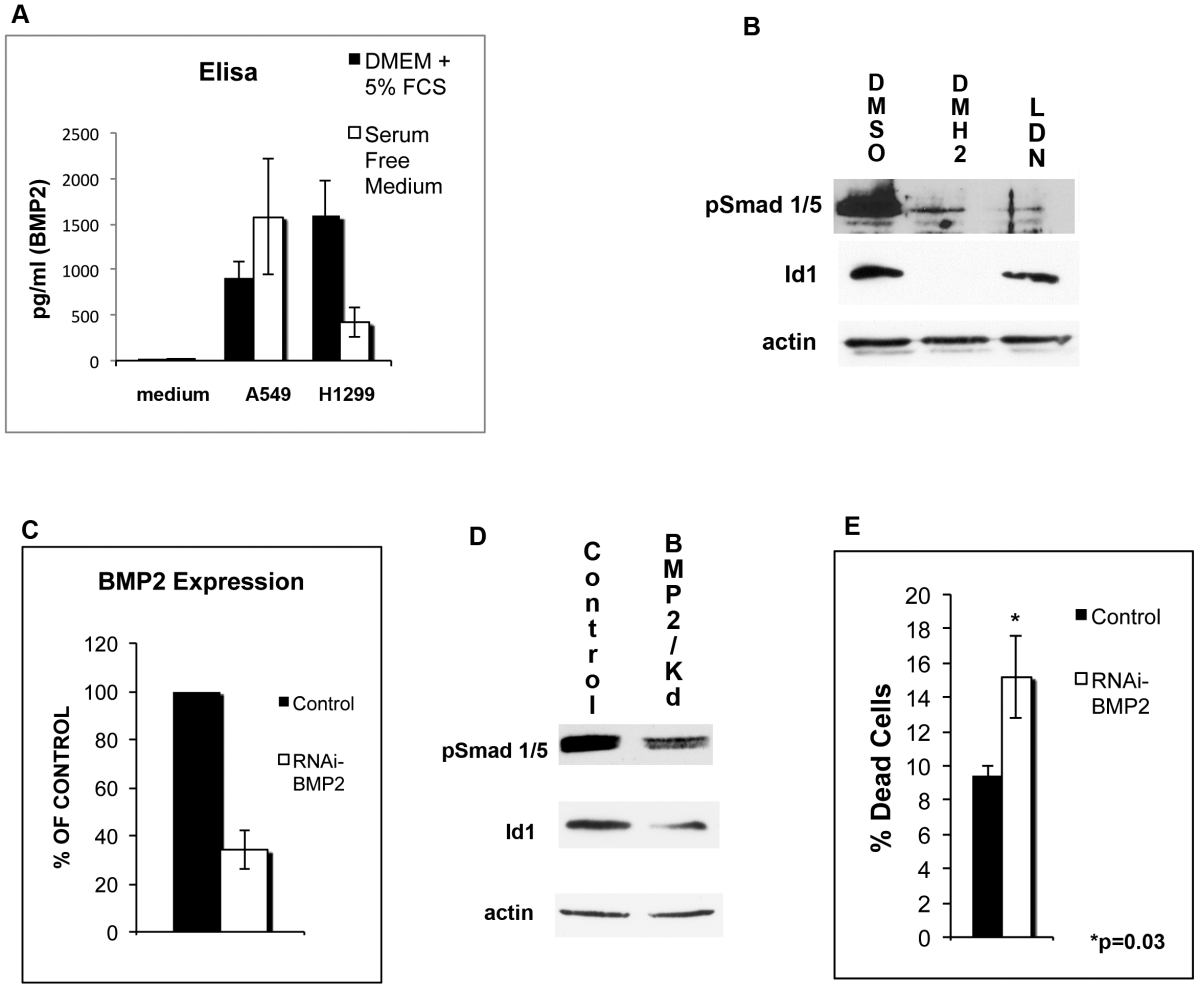
Lung cancer cell lines secrete BMP2 and knockdown of BMP2 decreases BMP signaling and induces cell death. (**A**) A BMP2 Elisa was performed on the DMEM 5% FCS and SFM in the absence of cells (medium). A BMP2 Elisa was performed on the DMEM 5% FCS and SFM cell culture medium containing A549 and H1299 cells for 48 hours. Experiments represent the mean of at least 5 experiments performed in triplicate. (**B**) BMP antagonists decrease BMP signaling of lung cancer cells cultured in SFM. H1299 cells cultured in SFM were treated with 1 µM DMSO, 1 µM of DMH2 or 1 µM of LDN for 48 hours and Western blot analysis performed. (**C**) Quantitative RT-PCR for BMP2 expression 48 hours after transfecting H1299 cells with control siRNA or siRNA targeting BMP2. Data represents the percent of control of the average of 4 independent experiments. (**D**) Western blot analysis of H1299 cells in SFM 48 hours after being transfected with control siRNA or siRNA targeting BMP2. BMP2 knockdown causes a decrease in expression of pSmad 1/5 and Id1. (**E**) Knockdown of BMP2 induces cell death. H1299 cells in SFM were transfected with control siRNA or siRNA targeting BMP2. After 48 hours the percentage of dead cells was determined by ethidium bromide staining. The data represents the mean of 4 independent experiments performed in triplicate.

The induction of cell death and growth suppression induced by BMP receptor antagonists of lung cancer cell lines growing in SFM suggested that BMP signaling occurs in a self-autonomous manner. Prior studies have shown that lung cancer cell lines produce the mature BMP2 protein, which is the active form [8]. To ensure that lung cancer cells secrete BMP2, an ELISA of the cell culture media was performed. BMP2 protein was not detected in DMEM with 5% fetal calf (FCS) or in serum free medium (SFM) (figure 6A). BMP2 was detected in the medium when lung cancer cell lines were cultured in either SFM or DMEM with 5% FCS (figure 6A). Since other BMPs could be in FCS, the effects of BMP receptor antagonists on the regulation of BMP signaling was examined in SFM. Phosphorylated Smad 1/5 and Id1 expression was detected in H1299 cells cultured in serum free medium (figure 6B). BMP type I receptor antagonists decreased pSmad 1/5 and Id1 expression of H1299 cells cultured in SFM (figure 6B).

To further examine self-autonomous signaling of the BMP signaling cascade, BMP2 expression was knocked down using siRNA. Quantitative RT-PCR showed that the siRNA targeting BMP2 caused a greater than 60% reduction in the expression of BMP2 (figure 6C). Knockdown of BMP2 of H1299 cells cultured in SFM caused a decrease in protein expression of phosphorylated Smad 1/5 and Id1 (figure 6D). BMP2 knockdown caused a significant increase in the percentage of dead lung cancer cells growing in SFM (figure 6E). These studies suggest that basal BMP activity of lung cancer cell lines is stimulated in a self-autonomous manner, which can be inhibited by antagonizing the BMP type I receptors.

### Knockdown of Id1 and Id3 Decreases Cell Growth and Induces Cell Death

**Figure 7:**
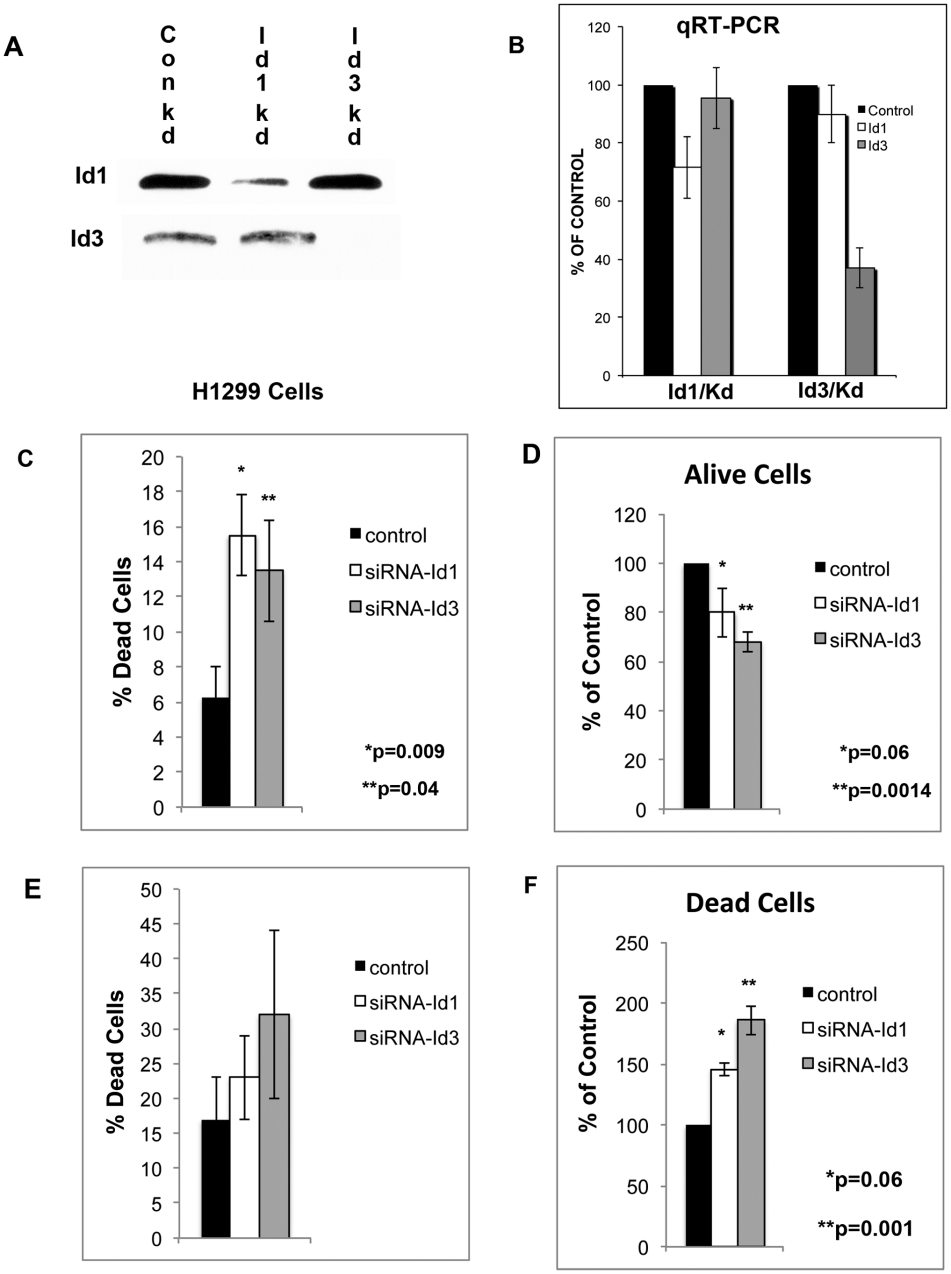
Id1 and Id3 regulate cell growth and survival of lung cancer cells. (**A**) Western blot analysis for Id1 and Id3 48 hours after H1299 cells were transfected with control siRNA and siRNA targeting Id1 or Id3. (**B**) Quantitative RT-PCR for Id1 and Id3 after H1299 cells were transfected with control siRNA and siRNA targeting Id1 or Id3**.** Data represents the percent control of the mean of 2 experiments. (**C**) H1299 cells were transfected with control siRNA or siRNA targeting Id1 or Id3. After 48 hours the percentage of cells staining for ethidium bromide was determined. The data is reported as the mean of 4 independent experiments. (**D–F**) H1299 cells were transfected with control siRNA and siRNA targeting Id1 or Id3. After 7 days the cells were stained with Trypan Blue and the number of alive and dead cells was determined. The data represents the percent change from siRNA control of (**D**) alive or (**F**) dead cells. (**E**) Represents the percentage of cells that were dead. The data represents the mean of 4 independent experiments.

Next, we examined whether Id1 and/or Id3 regulates cell survival and growth of lung cancer cells. Knockdown of Id1 using siRNA caused a reduction in the protein level of Id1 but not Id3 (figure 7A). Knockdown of Id3 caused a reduction of Id3 protein levels without effecting the expression of Id1 (figure 7A). Quantitative RT-PCR also showed a reduction in Id1 and Id3 respectively (figure 7B). Knockdown of Id1 or Id3 in H1299 cells caused a significant increase in the percentage of dead cells as determined by ethidium bromide staining (figure 7C). Cell counts using Trypan Blue staining showed that knockdown of Id1 decreased cell growth and induced cell death but did not reach statistical significance (figure 7D,F). Knockdown of Id3 did cause a highly significant decease in cell growth and induction of cell death when compared to controls (figure 7D,F). These studies suggest that reduction of Id expression is at least in part the mechanism by which BMP receptor antagonists induce cell death and decrease cell growth. In H1299 cells, Id3 may have a greater role in regulating BMP signaling than Id1.

### Cells Overexpressing Id3 are Resistant to DMH2

**Figure 8:**
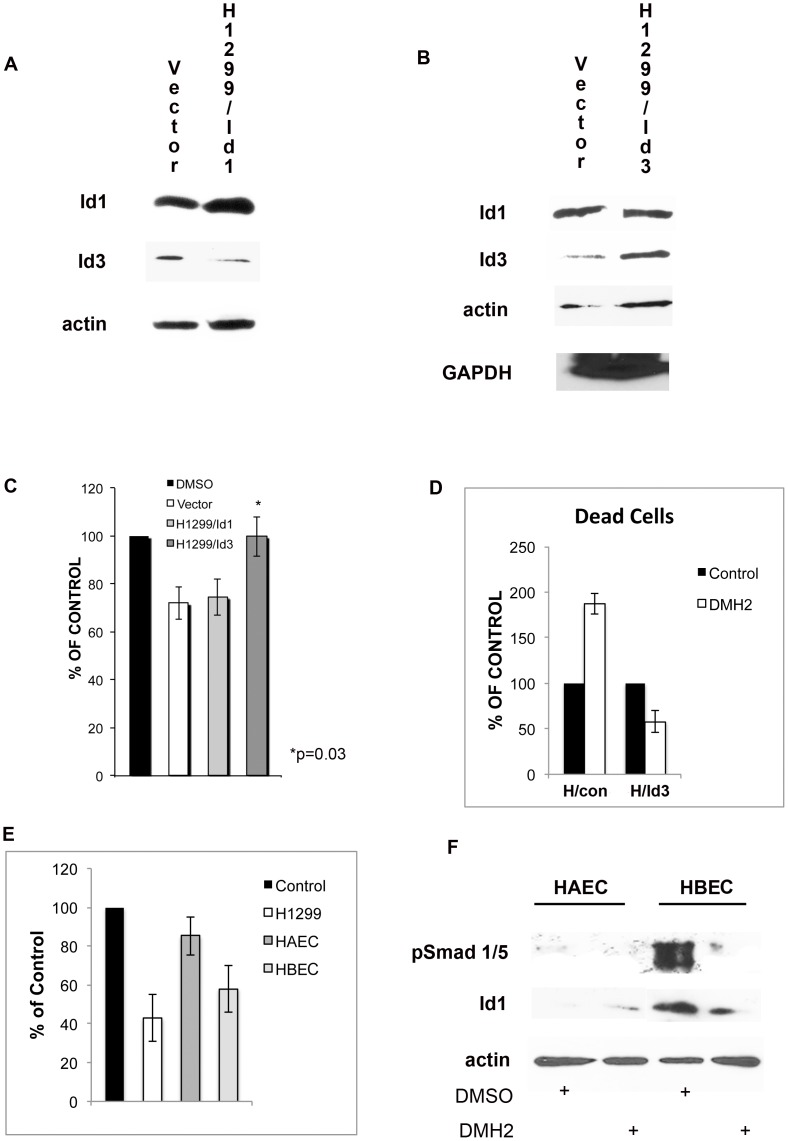
Forced expression of Id3 prevents growth suppression and cell death induced by DMH2. H1299 cells were stably transfected with Id1 and Id3 expression vectors or the insertless vector. (**A–B**) Western blot analysis showing increased expression of (**A**) Id1 or (**B**) Id3 in the transfected cell line. (**C–D**) H1299/Id1 and H1299/Id3 cells were treated with 1 µM DMSO or 1 µM DMH2 for 7 days and the percent alive and dead cells determined. (**C**) DMH2 caused growth suppression of vector control and H1299/Id1 cells but not H1299/Id3 cells. (**D**) DMH2 induced cell death in the vector control cells (H/con) but not in the H1299/Id3 cells (H/Id3). The data represents the mean of at least 3 experiments reported as the percent of control treated cells. (**E**) DMH2 deceases cell growth of immortalized normal human bronchial epithelial cells (HBEC) but not human aortic endothelial cells (HAEC). Cell lines growing in SFM were treated with 1 µM DMSO or 1 µM DMH2 for 7 day and cell counts performed. Data represents the mean of at least 3 experiments reported as the percent of control treated cells. (**F**) Western blot analysis showing higher expression of pSmad 1/5 and Id1 in HBEC compared to HAEC. 1 µM of DMH2 for 48 hours decreased expression of pSmad 1/5 and ID1 in HBEC but not HAEC.

To further define whether Id1 and Id3 mediate BMP signaling, Id1 and Id3 expression vectors were stably transfected into H1299 cells. Forced expression of Id1 caused an increase in protein expression of Id1 but not Id3 (figure 8A). Forced expression of Id3 caused an increase expression of Id3 but not Id1 (figure 8B). There was a small increase in actin expression in the H1299/Id3 cells. Since actin could be up regulated by Id3 overexpression, the expression of GAPDH was examined. By Western blot analysis demonstrated that GAPDH expression was the same between vector control cells and the H1299/Id3 cells. DMH2 caused a similar reduction in cell growth of the H1299/Id1 cells as compared to the vector control cells (figure 8C). DMH2 did not cause growth inhibition (figure 8C) or induce cell death (figure 8D) of the H1299/Id3 cells. These data further support that the biological effects mediated by BMP receptor antagonists involves Id proteins.

### DMH2 Suppresses Growth of Normal Bronchial Epithelial Cells but not Endothelial Cells

The BMP2 signaling cascade is essential for early lung development with high expression in epithelial and vascular progenitors [28]. At completion of lung morphogenesis BMP signaling declines with barely detectable expression in adult normal lung tissue [9], [28], [29]. BMP signaling is re-activated in normal adult bronchial epithelial cells by inflammation and tissue injury [28], [29]. To further define the specificity of BMP receptor antagonists, we examined whether normal human bronchial epithelial cells (HBEC) immortalized without viral oncoproteins [30] and normal human aortic endothelial cells (HAEC) are responsive to DMH2. As expected DMH2 caused a significant reduction in the growth of H1299 cells (57%) and a similar reduction in HBEC (42%) (figure 8E). DMH2 caused only a 15% reduction in cell growth of HAEC (figure 8E). HBEC had a higher level of expression of pSmad 1/5 and Id1 than HAEC (figure 8F). DMH2 caused a reduction in the expression of pSmad 1/5 and Id1 in HBEC but not in the HAEC. BMP antagonists also decreased Id1 expression and induced growth inhibition of the normal human bronchial epithelial cells immortalized with SV40 large T antigen gene (BEAS-2B cells) [31] (figure S4 and data not show). These studies further support that the biological effects induced by antagonists of the type I BMP receptor is from the inhibition of the BMP signaling cascade.

## Discussion

BMP family members are aberrantly expressed in many carcinomas including those arising from the lung, breast, prostate, ovarian, esophageal, and colon carcinomas [32], [33]. BMP-2 is expressed approximately 17 fold higher in NSCLC compared to normal lung or benign lung tumors [9]. BMP-4, BMP-6, and GDF-5 are also expressed in NSCLC but less frequently and by a lower amount than BMP-2 [9]. BMP-2, BMP-4, and GDF-5 bind to alk3 and alk6 with high affinity [1]. BMP-6 and BMP-7 bind more efficiently to alk2 and alk3 [1]. BMP-2/4 can also signal through alk2 and BMP-6/7 through alk6 [2]. Activation of the BMP receptors occurs from secreted ligands. Preformed type I and type II BMP receptor oligomers can also activate BMP signaling independent of a ligand [34]. Knockdown of BMP2 caused a biological response suggesting it is a major regulatory ligand in NSCLC. Since there is other BMP ligands present in lung cancer and possibly preformed receptor oligomers, antagonists of the type I receptors may be a good strategy to inhibit BMP signaling in cancer. Because there is little to no activity of the BMP signaling cascade in the epithelial cells of normal lung tissue, with reactivation occurring in inflamed, damaged, or transformed bronchial epithelial cells, suggests that there is a therapeutic window to target the BMP signaling pathway in lung cancer.

Our data suggests that BMP signaling is mediated in lung cancer cells through multiple BMP type I receptors. We show that alk2, alk3, and alk6 are expressed in lung cancer cell lines. A prior report showed that alk3 and alk6 are expressed in NSCLC [8]. The expression of alk2 in primary NSCLC has not been reported. Knockdown of a single BMP type I receptor was not sufficient to inhibit BMP signaling and its regulation of the downstream target Id1. Silencing of more than one type I BMP receptors was required to inhibit BMP signaling in lung cancer cell lines. DMH2, which caused the greatest inhibition of alk3 and alk6, also induced the greatest reduction in the expression of Id1. DMH2 is also reported to be a potent antagonist of alk2 [26]. In the H1299 cell line, inhibition of alk2 and alk3 was sufficient to reduce Id1 expression. There appeared to be cross regulation between the different receptors. Knockdown of alk2 and alk3 caused a reduction in expression of alk6. Knockdown of alk2, alk3, and alk6 also decreased Id1 protein expression in H1299 and A549 cells. These studies suggest that an antagonists targeting all function type I BMP receptors in cancer cells may cause the most significant suppression of BMP signaling. However, it is possible that an antagonist only targeting alk2 and alk3 could decrease alk6 expression leading to decreased BMP signaling. Since DMH2 is a selective BMP type I antagonist with very good in vitro activity it is an excellent candidate drug to study its efficacy in a lung tumor xenograft model.

Our studies support that the BMP signaling cascade is growth promoting in cancer. Antagonizing BMP type I receptors in lung cancer cell lines caused significant growth inhibition, decreased clonogenic growth, and induced cell death. BMPs having a growth-promoting role in cancer is consistent with its role as an essential growth enhancing morphogen during development [35] [36]. Prior studies suggesting that BMP signaling is growth suppressive or only has a minimal effect on cell growth may be explained by differences in study design [37], [38], [39], [40]. Recombinant BMP proteins produce a transient response in cell lines and are known to induce the expression of BMP antagonists [41]. The induction of BMP antagonists may attenuate any potential for an enhanced mitogenic response. Our studies suggest that the knockdown of a single BMP receptor may not cause a sufficient inhibition of BMP signaling in some cell lines. Blocking the basal activity of all functional BMP type I receptors effectively decreases signaling and has provided a new insight into the role of BMP signaling in cancer.

We show that the basal BMP activity in lung cancer cell lines is an essential regulator of Id1, Id2, and Id3 expression. Since the Id family members promote tumorigenesis in so many types of cancers, inhibiting their expression may have important therapeutic implications. Our studies suggested a greater role for Id3 in regulating BMP induced cell growth and survival of lung cancer cells than Id1. However, the Id family member regulating growth and survival of cancer cells may be tumor dependent. Numerous studies have reported that Id1 regulates growth and survival of lung and other tumors. The stimulation of cell growth, invasion, and metastasis has been attributed to Id1, Id2, and Id3 [42], [43]. Id4 is thought to act as a tumor suppressor [44], [45], [46]. In breast cancer, silencing both Id1 and Id3 caused a significantly greater reduction in tumor initiation and lung colonization than knockdown of either Id1 or Id3 alone [18]. The Human Protein Atlas database (www.proteinatlas.org) reports that Id1 is expressed more frequently in NSCLC than Id3. Therefore, the Id family mediating tumorigenesis may vary depending on which Id proteins are expressed.

Recent studies, using monoclonal antibodies, have suggested that the expression of Id family members is confined to a specific population of cancer cells. In breast cancer, Id1 and Id3 are expressed predominately in triple negative tumors (estrogen -, progesterone -, and Her2Neu-) [18]. Id1 is frequently over-expressed in NSCLC, occurring in 70% of squamous and 50% of adenocarcinomas [23]. Id2 is also over-expressed in most NSCLC [24]. The expression of Id3 has not been published but The Human Protein Atlas database reports that it is expressed in 36% of NSCLC. It is not know whether Id family members are expressed in a specific cell population in lung carcinomas. Reports have suggested that specific population of cancer cells have the capacity to self-renew. Since BMP signaling and Id family members regulate self-renewal and cell fate decisions of stem cells, it will be of interest to determine there role in the regulation of cancer cells with stem cell like characteristics. Further studies are needed to better characterize the expression of Id family members in NSCLC and determine in an animal model whether expression correlates with a response to BMP receptor antagonists.

We show that antagonizing BMP type I receptors leads to cell death. The mechanism by which inhibiting BMP signaling induces cell death was not revealed but does involve the down regulation of Id family members. During develop BMP signaling inhibits apoptosis of stem cells [5], [47], [48], [49]. BMP2 has also been shown to decrease hypoxic cell death of breast cancer cells [50]. Several studies have shown that Id1 inhibits apoptotic cell death of cancer cells [22], [51], [52], [53]. We did not detect the induction of apoptotic cell death by BMP receptor antagonists in our study. It is possible that since the percentage of cells that died was low, we were not able to detect apoptotic cell death by Western blot analysis. Other causes of cell death such as caspase independent cell death, necrosis, senescence, autophagy, and mitotic catastrophe are other potential mechanisms induced by BMP receptor inhibition [54].

The BMP signaling cascade is an essential regulator of the basal expression of Id family members in lung cancer cell lines. Our studies show that BMP signaling promotes cell growth, survival, and clonogenicity of lung cancer cells, which involves the regulation of Id family members. The growth promoting effects of BMP signaling can be inhibited by specific small molecule antagonists of the type I BMP receptors. BMP receptor antagonists may represent a novel means to treat lung and other cancers that depend on the BMP and/or Id family members to sustain tumor viability.

## Materials and Methods

### Plasmids

Constitutively active alk3 and alk6 constructs in mammalian vectors were a gift from Joan Massague (New York, New York) [55]. BRE-luciferase plasmid containing Smad 1/5/8 binding sites derived from the Id1 promoter [56] was a gift from Isaak Kim (UMDNJ Medical School). The Id expression vectors (PLXSN Id-1), (PLXSN Id-3) and control vector (PLXSN) was a gift from Pierre Desprez (California Pacific Medical Center).

### Cell Culture and Reagents

The cell lines, A549 and H1299 lung cancer cell lines were cultured in Dulbecco’s Modified Eagle’s medium (DMEM, Sigma Aldrich, St Louis, MO, USA) with 5% fetal bovine serum (FBS) containing 1% penicillin/streptomycin, and 1% glutamine. The A549 and H1299 cell lines were obtained from ATCC. Human bronchial epithelial cells immortalized with cyclin dependent kinase 4 (CdK4) and human telomerase reverse transcriptase (hTERT) [30] or SV40 large antigen [57] were cultured in SFM. Human aortic endothelial cells (Invitrogeon) were also cultured in SFM. Cells were kept in a humidified incubator with 5% CO2 at 37°C [58]. Serum free medium (LHC-9, Life Technology, Grand Island, MI) was also used in specified experiments. In experiments using SFM, medium containing FCS was replaced with SFM approximately 24 hours prior to the experiment. Dorsomorphin (compound C) was purchased from Sigma. Dorsomporphin analogues DMH1, DMH2, and LDN were a kind gift from Charles Hong (Vanderbelt University). Dorsomorphin is a small molecule antagonist of the BMP type I receptors [59]. Dorsomorphin analogues DMH1, DMH2, [60] and LDN [61] are more specific and potent antagonists of the type I BMP receptors. LDN has less activity toward AMP kinase than Dorsomorphin (32). DMH1 and DMH2 are even more specific analogues that have less activity toward VEGF II, AMP kinase, TGFβ receptor alk5, and platelet-derived growth factor receptor-β than Dorsomorphin and LDN (33).

### Quantification of Gene Expression

RNA was extracted using the RNeasy kit as per the manufacturer's instructions (Qiagen, Valencia, CA). DNAase was used to remove any DNA contamination. cDNA was generated using Advantage RT for PCR kit (BD BiosciencesClontech, Palo Alto, CA). Quantitative PCR was performed with the Stratagene Mx3005p (Agilent Technologies) and predesigned validated Taq-Man gene expression assays according to the manufacturer’s specifications (Applied Biosystems, Foster City, CA). Reference numbers used are: GAPDH (Hs99999905_m1), ACVRL1 (alk1) (Hs00163543_m1), ACVR1A (alk2) (Hs00153836_m1), BMR1A (alk3) (Hs00831730_s1), BMPR1B (alk6) (Hs00176144_m1), Id1 (Hs00357821_g1), Id2 (Hs00747379_m1), and Id3 (Hs00171409-m1). Primers for BMP2 were (F)-5-CCT-GAG-CGA-GTT-CGA-GTT-G-3 and (R) 5-CAC-TCG-TTT-CTG-GTA-GTT-C-3. Syber green was used for BMP2 primers (Qiagen, Germantown, MD). Negative control included all reagents except cDNA. Expression was normalized to GAPDH or actin using the formula 2^Δ CT^.

### Transient Gene Knockdown

Two sets of Silencer Select Validated and Pre-designed siRNA were used to target the type I BMP receptors alk2, alk3, and alk6 (Life Technologies). The ID numbers to the first set of siRNA are: alk2 (s974-validated), alk3 (s281-Pre-designed), alk6 (s2042-Pre-designed). The siRNA ID numbers for the second set of siRNA are: alk2 (s975-validated), alk3 (s280-Pre-designed), and alk 6 (s2041-Pre-designed). Silencer Select ID numbers used for Id1 and Id3 knockdowns are: Id1 (s7106- Pre-designed), Id3 (s7111-Pre-designed). The Id number for BMP2 is s2020 (Pre-designed). Silencer Select Negative Control siRNA (4390843) was used to confirm specificity of each targeted knockdown.

A549 and H1299 cells were transfected with siRNA using a Nucleofector II (Amaxa Biosystems, Gaitherburg, MD) using the manufacture’s Nucleofector kit T. Optimization was performed using the enhanced green fluorescent reporter (EGFP) (Clontech) expressed in the pcDNA 3.1 vector (Invitrogen), which showed approximately 80% of the cells were transfected using this transfection protocol. A total of 30 nM of siRNA was used for alk2 and alk3. For alk6, Id1, Id3, and BMP2 a total of 20 nM of siRNA was used. An equal amount of control siRNA was used in each experiment. The siRNA was delivered to 1×10^6^ A549 and H1299 cells and cultured for 48 hours in DMEM with 5% FCS. BMP receptors and Id1 expression was measured by qPCR.

### Western Blot Analysis

Total cellular protein was prepared using RIPA buffer containing a protease inhibitor cocktail and protein concentration was measured using the BCA assay as described [8]. In brief, protein was analyzed by SDS-PAGE, transferred to nitrocellulose (Schleicher and Schuell, Keene, NH). After blocking, the blots were incubated overnight at 4°C with the appropriate primary antibody in Tris-buffered saline with 1% Tween (TBST) and 5% non-fat milk. Secondary antibodies were applied for 1 hour at room temperature. Specific proteins were detected using the enhanced chemiluminescence system (Amersham, Arlington Heights, IL). The primary antibodies that were used were rabbit monoclonal anti-pSmad 1/5/8 (Cell signaling Technology, Danvers MA) rabbit anti-actin, an affinity isolated antigen specific antibody (Sigma, Saint Louis, MO), rabbit monoclonal anti-Id1 and rabbit monoclonal anti-Id3 (Calbioreagents, San Mateo, CA).

### Luciferase Assay

10^6^ H1299 cells were transfected with 2 µg of BRE-luciferace plasmid using a Nucleofector II. Fourty-eight hours later the cells were treated with DMSO or a BMP receptor antagonist and cell lysates were harvested 24 hours after treatment. Cells were lysed with luciferase lysis buffer (Promega). Samples were added to luciferase assay substrate (Promega) and luminsescence measured by the TD-20/20 Luminometer (Turner Designs/Turner BioSystems, Sunnyvale, CA). Control samples included luciferase assay substrate alone and luciferase assay substrate plus 1× reporter lysis buffer.

To assess the effects of antagonists on a specific type I BMP receptor, 2 µg of constitutively active ca-alk3 and ca-alk6 expression vectors were co-transfected with the BRE-luciferase reporter into H1299 cells with Nucleofector II. Control cells were co-transfected with the pcDNA3 expression vector. After 48 hours the cells were treated with DMSO or a BMP receptor antagonist for 48 hours and luminescence was measured.

To examine the effects of knockdown of a single type I BMP receptor on Smad 1/5/8 activity, H1299 cells were co-transfected with siRNA targeting alk2, alk3, or alk6 together with the BRE-luciferase reporter. Luminescence was measured 48 hours after transfection.

### Cell Death Assay

A549 and H1299 cells were plated in 6 well plates with 10^6^ cells per well. Cells were treated with DMSO or a BMP receptor antagonist for 12 and 48 hours. Adherent and floating cells were harvested and incubated with 0.1 mg/ml of ethidium bromide. Immediately after staining approximately 100 cells were counted and the percentage of cells that took up ethidium bromide was determined.

Cell death was also determined using the LIVE/DEAD fixable dead cell stain kit as per manufacturer’s instructions (Life Technologies, L-23101). This assay employs an amine-reactive fluorescent dye, which in compromised membranes the dye reacts with free amines on the cell interior in dead cells. H1299 cells were treated with DMH2 for 48 hours and the percentage of dead cells then detected by flow cytometry.

Cell death was also determined by treating H1299 cells, cultured in DMEM 5% FCS, with DMSO, DMH2 1 µM, and DMH2 5 µM for 7 days, floating and adherent cells were stained with Trypan Blue and cell counts performed. The percentage of live and dead cells was then determined. Cell counts and Trypan Blue staining was also done on 1299 cells cultured in SFM and treated with 1 µM DMSO or 1 µM DMH2 for 7 days.

Knockdown of all type I BMP receptors was performed in the H1299 cells by transfecting siRNA for alk2, alk3, and alk6 or control siRNA. Two days after the transfection the cells were harvested and the percentage of cells staining for ethidium bromide was determined.

### Apoptosis

To detect apoptosis H1299 cells were treated with DMSO or 1 µM DMH2 for 24, 48, and 72 hours. The cleaved activated Caspase-3 fragment was assessed by Western Blot analysis (Cell Signaling). Cells treated with Staurosporine were used as a positive control. Morphological changes of apoptosis such as cell shrinkage and chromatin condensation was examined by video microscopy of H1299 cells treated with 1 µM of LDN, DMH1, or DMH2 between 24 to 48 hours following treatment.

### Clonigenic Growth Assay

A549 and H1299 cells were plated into 6 well plates with 500 cells per well. The next day the cells were treated with DMSO or a BMP receptor antagonist for 2 weeks. The colonies were stained with Diff-Quick (IMEB Inc. San Marcos, CA) and the total number of colonies per well counted.

### Cell Counts

A549 and H1299 cells were plated into 6 well plates with 100,000 cells per well. Cells were treated with DMSO or a BMP receptor antagonist for 7 days. The cells were detached with trypsin, stained with trypan blue, and the number of live cells counted using a hemacytometer.

### BrdU Assay

Thirty-thousand H1299 cells were plated into 96 well plates. The next day the cells were treated with 1 µM DMSO, 5 µM DMSO, 1 µM DMH2, or 5 µM DMH2 for 24 and 48 hours. BrdU incorporation was measured using the Cell Proliferation ELISA, BrdU colorimetric kit as per manufacture’s instruction (Roche, Indianapolis, OH). Cells were incubated with BrdU labeling solution for 2 hours, fixed/denatured, and BrdU located with a peroxidase-conjugated anti-BrdU antibody. Knockdown of all type I BMP receptors were performed in the H1299 cells by transfecting siRNA for alk2, alk3, and alk6 or control siRNA. Three days after transfection BrdU incorporation was determined. Studies were performed 4 times in triplicate.

### Soft Agar Assay

A 1% agar mixture was prepared in sterile double-distilled water, microwaved, and cooled to 40°C in a water bath. DMEM was also incubated at 40°C. Equal amounts of each were mixed and 1 ml added to each well of a six well plate. The base agar was allowed to solidify. A 0.7% agar mixture was prepared then cooled to 40° degrees. Cells were trypsinized and counted. Cells (2500 cells per well) were placed in pre-warmed DMEM containing DMSO or DMH1 (1 µM/ml). The treated cells were then mixed with the 0.7% agar and 2 ml placed on top of the base agar. Once solidified, 1 ml of DMEM was placed on top of the agar. Colonies were counted four weeks later using a microscope.

### BMP2 Elisa

H1299 and A549 cells were seeded into 6 well plates in duplicate at 150,000 cells/well. The cells were incubated for 4 days in either DMEM 5% FCS or SFM. Cell culture medium as also placed into wells that did not contain cells. The medium was collected and BMP2 Elisa performed as per manufacture’s instructions (PeproTech, Rocky Hill, NJ).

### Statistical Analysis

The mean of the control group was compared to the mean of each treated group using a paired student t-test assuming unequal variances. Differences with p values ≤.05 were considered statistically significant.

## Supporting Information

Figure S1 **DMH2 decreases phosphorylated Smad 1/5/8 expression in A549 cells.** Western blot analysis for pSmad 1/5/8 on A549 cells treated with 1 µM DMSO, DMH1 or DMH2 for 12, 24, and 24 hours.
(TIF)

Figure S2 **Dorsomorphin and LDN decrease protein expression of Id1 and Id3.** Western blot analysis for Id1 and Id3 on A549 cells treated with 10 µM Dorsomorphin or 1 µM LDN for 24 and 48 hours.
(TIF)

Figure S3 **Knockdown of multiple BMP type I receptors using a second set of siRNA decreases BMP signaling, decreases proliferation, and induces cell death.** **(A–B)** A549 and H1299 cells were transfected with siRNA targeting each type I BMP receptor and quantitative RT-PCR was performed for that BMP receptor. **(C)** Knockdown of each BMP type I receptor in H1299 cells was performed and quantitative RT-PCR performed for all 3 type I receptors. **(D–E)** A549 and H1299 cells were transfected with siRNA targeting a single type I BMP receptor or siRNA control. After 48 hours quantitative RT-PCR was performed for Id1. **(F)** H1299 cells were co-tranfected with BRE-luciferase reporter and siRNA for a single type I BMP receptor. After 48 hours luciferace activity was measured. **(G)** Knockdown of all type I BMP receptors was performed in A549 cells. Quantitative RT-PCR showed significant reduction in Id1 expression. **(H)** Quantitative RT-PCR shows a reduction of all 3 BMP type I receptors. **(I)** Western blot analysis for Id1 in H1299 cells with knockdown of a single type I BMP receptor or combination knockdown of alk2 and alk3, or all 3 BMP type I receptors. Studies show silencing more than one receptor is required to decrease Id1 expression. **(J)** Transfection of H1299 cells with siRNA targeting all type I receptors causes significant reduction of alk2 and alk6 with a corresponding significant reduction in **(K)** proliferation and **(L)** induction of cell death. **(B,C,D,E,G,H,J,K)** Data represents the mean of at least 3 experiments reported as the percent of control treated cells. **(F,L)** Data represents the mean of at least 3 experiments.
(TIF)

Figure S4 **Western blot analysis showing immortalized normal human bronchial epithelial (BEAS-2B) cells treated with BMP receptor antagonists causes a significant reduction in the expression of Id1 and Id3.**
(TIF)

## References

[1] Nickel J, Sebald W, Groppe JC, Mueller TD (2009) Intricacies of BMP receptor assembly. Cytokine Growth Factor Rev 20: 367–377. 19926516 10.1016/j.cytogfr.2009.10.022

[2] Lavery K, Swain P, Falb D, Alaoui-Ismaili MH (2008) BMP-2/4 and BMP-6/7 differentially utilize cell surface receptors to induce osteoblastic differentiation of human bone marrow-derived mesenchymal stem cells. J Biol Chem 283: 20948–20958. 18436533 10.1074/jbc.M800850200 PMC3258927

[3] Attisano L, Wrana JL (2002) Signal transduction by the TGF-beta superfamily. Science 296: 1646–1647. 12040180 10.1126/science.1071809

[4] Pizette S, Abate-Shen C, Niswander L (2001) BMP controls proximodistal outgrowth, via induction of the apical ectodermal ridge, and dorsoventral patterning in the vertebrate limb. Development 128: 4463–4474. 11714672 10.1242/dev.128.22.4463

[5] Yang X, Castilla LH, Xu X, Li C, Gotay J, et al (1999) Angiogenesis defects and mesenchymal apoptosis in mice lacking SMAD5. Development 126: 1571–1580. 10079220 10.1242/dev.126.8.1571

[6] Chang H, Huylebroeck D, Verschueren K, Guo Q, Matzuk MM, et al (1999) Smad5 knockout mice die at mid-gestation due to multiple embryonic and extraembryonic defects. Development 126: 1631–1642. 10079226 10.1242/dev.126.8.1631

[7] Ying QL, Nichols J, Chambers I, Smith A (2003) BMP induction of Id proteins suppresses differentiation and sustains embryonic stem cell self-renewal in collaboration with STAT3. Cell 115: 281–292. 14636556 10.1016/s0092-8674(03)00847-x

[8] Langenfeld EM, Calvano SE, Abou-Nukta F, Lowry SF, Amenta P, et al.. (2003) The mature bone morphogenetic protein-2 is aberrantly expressed in non-small cell lung carcinomas and stimulates tumor growth of A549 cells. Carcinogenesis 24: 1445–1454. Epub 2003 Jun 1419. 10.1093/carcin/bgg100 12819188

[9] Langenfeld EM, Bojnowski J, Perone J, Langenfeld J (2005) Expression of bone morphogenetic proteins in human lung carcinomas. Ann Thorac Surg 80: 1028–1032. 16122479 10.1016/j.athoracsur.2005.03.094

[10] Le Page C, Puiffe ML, Meunier L, Zietarska M, de Ladurantaye M, et al (2009) BMP-2 signaling in ovarian cancer and its association with poor prognosis. J Ovarian Res 2: 4. 19366455 10.1186/1757-2215-2-4 PMC2674440

[11] Yuen HF, Chan YP, Cheung WL, Wong YC, Wang X, et al.. (2008) The prognostic significance of BMP-6 signaling in prostate cancer. Mod Pathol 21: 1436–1443. Epub 2008 Oct 1417. 10.1038/modpathol.2008.94 18931653

[12] Park Y, Kang MH, Seo HY, Park JM, Choi CW, et al (1192) Bone morphogenetic protein-2 levels are elevated in the patients with gastric cancer and correlate with disease progression. Med Oncol 27: 1192–1199. 10.1007/s12032-009-9358-x 19924575

[13] Langenfeld EM, Langenfeld J (2004) Bone morphogenetic protein-2 stimulates angiogenesis in developing tumors. Mol Cancer Res 2: 141–149. 15037653

[14] Raida M, Clement JH, Leek RD, Ameri K, Bicknell R, et al.. (2005) Bone morphogenetic protein 2 (BMP-2) and induction of tumor angiogenesis. J Cancer Res Clin Oncol 131: 741–750. Epub 2005 Nov 2001. 10.1007/s00432-005-0024-1 PMC12161192 16136355

[15] Rothhammer T, Bataille F, Spruss T, Eissner G, Bosserhoff AK (2007) Functional implication of BMP4 expression on angiogenesis in malignant melanoma. Oncogene 26: 4158–4170. Epub 2006 Dec 4118. 10.1038/sj.onc.1210182 17173062

[16] Langenfeld EM, Kong Y, Langenfed J (2005) Bone morphogenetic protein 2 stimulation of tumor growth involves the activation of Smad-1/5. Oncogene Epub. 10.1038/sj.onc.1209110 16247476

[17] Alani RM, Young AZ, Shifflett CB (2001) Id1 regulation of cellular senescence through transcriptional repression of p16/Ink4a. Proc Natl Acad Sci U S A 98: 7812–7816. Epub 2001 Jun 7826. 10.1073/pnas.141235398 PMC35424 11427735

[18] Gupta GP, Perk J, Acharyya S, de Candia P, Mittal V, et al (2007) ID genes mediate tumor reinitiation during breast cancer lung metastasis. Proc Natl Acad Sci U S A 104: 19506–19511. 18048329 10.1073/pnas.0709185104 PMC2148319

[19] Swarbrick A, Roy E, Allen T, Bishop JM (2008) Id1 cooperates with oncogenic Ras to induce metastatic mammary carcinoma by subversion of the cellular senescence response. Proc Natl Acad Sci U S A 105: 5402–5407. Epub 2008 Mar 5431. 10.1073/pnas.0801505105 PMC2291136 18378907

[20] Ling MT, Wang X, Zhang X, Wong YC (2006) The multiple roles of Id-1 in cancer progression. Differentiation 74: 481–487. 17177845 10.1111/j.1432-0436.2006.00083.x

[21] Swarbrick A, Akerfeldt MC, Lee CS, Sergio CM, Caldon CE, et al (2005) Regulation of cyclin expression and cell cycle progression in breast epithelial cells by the helix-loop-helix protein Id1. Oncogene 24: 381–389. 15489884 10.1038/sj.onc.1208188

[22] Li B, Tsao SW, Li YY, Wang X, Ling MT, et al (2009) Id-1 promotes tumorigenicity and metastasis of human esophageal cancer cells through activation of PI3K/AKT signaling pathway. Int J Cancer 125: 2576–2585. 19551863 10.1002/ijc.24675

[23] Rothschild SI, Kappeler A, Ratschiller D, Betticher DC, Tschan MP, et al. The stem cell gene "inhibitor of differentiation 1" (ID1) is frequently expressed in non-small cell lung cancer. Lung 71: 306–311. Epub 2010 Aug 2014. 10.1016/j.lungcan.2010.06.018 20709421

[24] Rollin J, Blechet C, Regina S, Tenenhaus A, Guyetant S, et al.. (2009) The intracellular localization of ID2 expression has a predictive value in non small cell lung cancer. PLoS One 4: e4158. Epub 2009 Jan 4158. 10.1371/journal.pone.0004158 PMC2612745 19129913

[25] Ponz-Sarvise M, Nguewa PA, Pajares MJ, Agorreta J, Lozano MD, et al. Inhibitor of Differentiation-1 as a Novel Prognostic Factor in NSCLC Patients with Adenocarcinoma Histology and Its Potential Contribution to Therapy Resistance. Clin 17: 4155–4166. Epub 2011 May 4153. 10.1158/1078-0432.CCR-10-3381 21540238

[26] Hao J, Ho JN, Lewis JA, Karim KA, Daniels RN, et al. In vivo structure-activity relationship study of dorsomorphin analogues identifies selective VEGF and BMP inhibitors. ACS Chem Biol 5: 245–253. 20020776 10.1021/cb9002865 PMC2825290

[27] Leite M, Quinta-Costa M, Leite PS, Guimaraes JE (1999) Critical evaluation of techniques to detect and measure cell death–study in a model of UV radiation of the leukaemic cell line HL60. Anal Cell Pathol 19: 139–151. 10866276 10.1155/1999/176515 PMC4618583

[28] Sountoulidis A, Stavropoulos A, Giaglis S, Apostolou E, Monteiro R, et al.. (2012) Activation of the Canonical Bone Morphogenetic Protein (BMP) Pathway during Lung Morphogenesis and Adult Lung Tissue Repair. PLoS One 7: e41460. Epub 42012 Aug 41420. 10.1371/journal.pone.0041460 PMC3423416 22916109

[29] Rosendahl A, Pardali E, Speletas M, Ten Dijke P, Heldin CH, et al (2002) Activation of bone morphogenetic protein/Smad signaling in bronchial epithelial cells during airway inflammation. Am J Respir Cell Mol Biol 27: 160–169. 12151307 10.1165/ajrcmb.27.2.4779

[30] Ramirez RD, Sheridan S, Girard L, Sato M, Kim Y, et al (2004) Immortalization of human bronchial epithelial cells in the absence of viral oncoproteins. Cancer Res 64: 9027–9034. 15604268 10.1158/0008-5472.CAN-04-3703

[31] Ke Y, Reddel RR, Gerwin BI, Miyashita M, McMenamin M, et al (1988) Human bronchial epithelial cells with integrated SV40 virus T antigen genes retain the ability to undergo squamous differentiation. Differentiation 38: 60–66. 2846394 10.1111/j.1432-0436.1988.tb00592.x

[32] Kiesslich T, Berr F, Alinger B, Kemmerling R, Pichler M, et al. Current Status of Therapeutic Targeting of Developmental Signalling Pathways in Oncology. Curr Pharm Biotechnol 2011: 24. 10.2174/138920112802502114 21605074

[33] Ye L, Mason MD, Jiang WG Bone morphogenetic protein and bone metastasis, implication and therapeutic potential. Front 16: 865–897. 10.2741/3725 21196208

[34] Nohe A, Hassel S, Ehrlich M, Neubauer F, Sebald W, et al.. (2002) The mode of bone morphogenetic protein (BMP) receptor oligomerization determines different BMP-2 signaling pathways. J Biol Chem 277: 5330–5338. Epub 2001 Nov 5319. 10.1074/jbc.M102750200 11714695

[35] Lawson KA, Dunn NR, Roelen BA, Zeinstra LM, Davis AM, et al (1999) Bmp4 is required for the generation of primordial germ cells in the mouse embryo. Genes Dev 13: 424–436. 10049358 10.1101/gad.13.4.424 PMC316469

[36] Kishimoto Y, Lee KH, Zon L, Hammerschmidt M, Schulte-Merker S (1997) The molecular nature of zebrafish swirl: BMP2 function is essential during early dorsoventral patterning. Development 124: 4457–4466. 9409664 10.1242/dev.124.22.4457

[37] Tada A, Nishihara T, Kato H (1998) Bone morphogenetic protein 2 suppresses the transformed phenotype and restores actin microfilaments of human lung carcinoma A549 cells. Oncol Rep 5: 1137–1140. 9683824 10.3892/or.5.5.1137

[38] Soda H, Raymond E, Sharma S, Lawrence R, Cerna C, et al (1998) Antiproliferative effects of recombinant human bone morphogenetic protein-2 on human tumor colony-forming units. Anticancer Drugs 9: 327–331. 9635923 10.1097/00001813-199804000-00006

[39] Beck SE, Jung BH, Del Rosario E, Gomez J, Carethers JM (2007) BMP-induced growth suppression in colon cancer cells is mediated by p21WAF1 stabilization and modulated by RAS/ERK. Cell Signal 19: 1465–1472. 17317101 10.1016/j.cellsig.2007.01.017 PMC3444522

[40] Miyazaki H, Watabe T, Kitamura T, Miyazono K (2004) BMP signals inhibit proliferation and in vivo tumor growth of androgen-insensitive prostate carcinoma cells. Oncogene 23: 9326–9335. 15531927 10.1038/sj.onc.1208127

[41] Langenfeld EM, Kong Y, Langenfeld J (2006) Bone morphogenetic protein 2 stimulation of tumor growth involves the activation of Smad-1/5. Oncogene 25: 685–692. 16247476 10.1038/sj.onc.1209110

[42] Shepherd TG, Theriault BL, Nachtigal MW (2008) Autocrine BMP4 signalling regulates ID3 proto-oncogene expression in human ovarian cancer cells. Gene 414: 95–105. Epub 2008 Mar 2004. 10.1016/j.gene.2008.02.015 18372118

[43] Gray MJ, Dallas NA, Van Buren G, Xia L, Yang AD, et al.. (2008) Therapeutic targeting of Id2 reduces growth of human colorectal carcinoma in the murine liver. Oncogene 27: 7192–7200. Epub 2008 Sep 7122. 10.1038/onc.2008.356 PMC3199128 18806828

[44] Chen SS, Claus R, Lucas DM, Yu L, Qian J, et al. Silencing of the inhibitor of DNA binding protein 4 (ID4) contributes to the pathogenesis of mouse and human CLL. Blood 117: 862–871. Epub 2010 Nov 2022. 10.1182/blood-2010-05-284638 PMC3035078 21098398

[45] Noetzel E, Veeck J, Horn F, Hartmann A, Knuchel R, et al (2008) [Promoter methylation of ID4. A marker for recurrence-free survival in human breast cancer]. Pathologe 29: 319–327. 18807039 10.1007/s00292-008-1038-7

[46] Umetani N, Mori T, Koyanagi K, Shinozaki M, Kim J, et al (2005) Aberrant hypermethylation of ID4 gene promoter region increases risk of lymph node metastasis in T1 breast cancer. Oncogene 24: 4721–4727. 15897910 10.1038/sj.onc.1208538

[47] Zhang J, Li L (2005) BMP signaling and stem cell regulation. Dev Biol 284: 1–11. 15963490 10.1016/j.ydbio.2005.05.009

[48] Izumi M, Fujio Y, Kunisada K, Negoro S, Tone E, et al (2001) Bone morphogenetic protein-2 inhibits serum deprivation-induced apoptosis of neonatal cardiac myocytes through activation of the Smad1 pathway. J Biol Chem 276: 31133–31141. 11408477 10.1074/jbc.M101463200

[49] Sugimori K, Matsui K, Motomura H, Tokoro T, Wang J, et al (2005) BMP-2 prevents apoptosis of the N1511 chondrocytic cell line through PI3K/Akt-mediated NF-kappaB activation. J Bone Miner Metab 23: 411–419. 16261446 10.1007/s00774-005-0622-7

[50] Raida M, Clement JH, Ameri K, Han C, Leek RD, et al (2005) Expression of bone morphogenetic protein 2 in breast cancer cells inhibits hypoxic cell death. Int J Oncol 26: 1465–1470. 15870857

[51] Mern DS, Hasskarl J, Burwinkel B (2010) Inhibition of Id proteins by a peptide aptamer induces cell-cycle arrest and apoptosis in ovarian cancer cells. Br J Cancer 103: 1237–1244. Epub 2010 Sep 1214. 10.1038/sj.bjc.6605897 PMC2967066 20842131

[52] Mern DS, Hoppe-Seyler K, Hoppe-Seyler F, Hasskarl J, Burwinkel B (2010) Targeting Id1 and Id3 by a specific peptide aptamer induces E-box promoter activity, cell cycle arrest, and apoptosis in breast cancer cells. Breast Cancer Res Treat 124: 623–633. Epub 2010 Feb 2027. 10.1007/s10549-010-0810-6 20191379

[53] Lin J, Guan Z, Wang C, Feng L, Zheng Y, et al.. (2010) Inhibitor of differentiation 1 contributes to head and neck squamous cell carcinoma survival via the NF-kappaB/survivin and phosphoinositide 3-kinase/Akt signaling pathways. Clin Cancer Res 16: 77–87. Epub 2009 Dec 2022. 10.1158/1078-0432.CCR-08-2362 PMC3321741 20028744

[54] Okada H, Mak TW (2004) Pathways of apoptotic and non-apoptotic death in tumour cells. Nat Rev Cancer 4: 592–603. 15286739 10.1038/nrc1412

[55] Zou H, Wieser R, Massague J, Niswander L (1997) Distinct roles of type I bone morphogenetic protein receptors in the formation and differentiation of cartilage. Genes Dev 11: 2191–2203. 9303535 10.1101/gad.11.17.2191 PMC275391

[56] Korchynskyi O, ten Dijke P (2002) Identification and functional characterization of distinct critically important bone morphogenetic protein-specific response elements in the Id1 promoter. J Biol Chem 277: 4883–4891. 11729207 10.1074/jbc.M111023200

[57] Ke Y, Reddel RR, Gerwin BI, Miyashita M, McMenamin M, et al (1988) Human bronchial epithelial cells with integrated SV40 virus T antigen genes retain the ability to undergo squamous differentiation. Differentiation 38: 60–66. 2846394 10.1111/j.1432-0436.1988.tb00592.x

[58] Langenfeld EM, Kong Y, Langenfeld J (2005) Bone morphogenetic protein-2-induced transformation involves the activation of mammalian target of rapamycin. Mol Cancer Res 3: 679–684. 16380505 10.1158/1541-7786.MCR-05-0124

[59] Yu PB, Hong CC, Sachidanandan C, Babitt JL, Deng DY, et al.. (2008) Dorsomorphin inhibits BMP signals required for embryogenesis and iron metabolism. Nat Chem Biol 4: 33–41. Epub 2007 Nov 2018. 10.1038/nchembio.2007.54 PMC2727650 18026094

[60] Hao J, Daleo MA, Murphy CK, Yu PB, Ho JN, et al (2008) Dorsomorphin, a selective small molecule inhibitor of BMP signaling, promotes cardiomyogenesis in embryonic stem cells. PLoS One 3: e2904. 18682835 10.1371/journal.pone.0002904 PMC2483414

[61] Yu PB, Deng DY, Lai CS, Hong CC, Cuny GD, et al.. (2008) BMP type I receptor inhibition reduces heterotopic [corrected] ossification. Nat Med 14: 1363–1369. Epub 2008 Nov 1330. 10.1038/nm.1888 PMC2846458 19029982

