# Supplementary figures and images for: Bone Morphogenetic Protein Type I Receptor Antagonists Decrease Growth and Induce Cell Death of Lung Cancer Cell Lines
Source: PLoS One. 2013 Apr 12;8(4):e61256. doi: 10.1371/journal.pone.0061256 (PMC3625205; doi:10.1371/journal.pone.0061256)

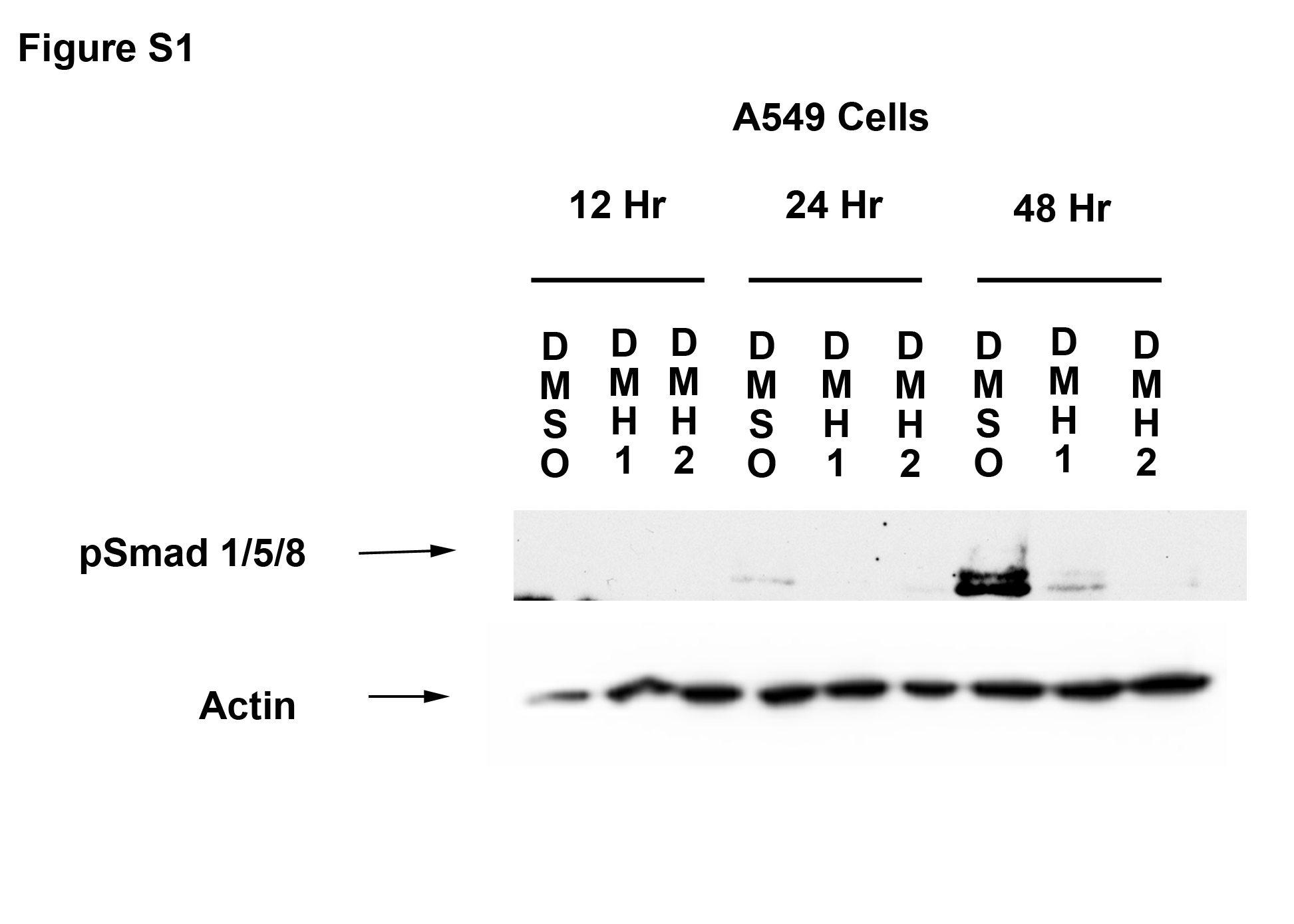

Supplement: Figure S1 — DMH2 decreases phosphorylated Smad 1/5/8 expression in A549 cells. Western blot analysis for pSmad 1/5/8 on A549 cells treated with 1 µM DMSO, DMH1 or DMH2 for 12, 24, and 24 hours. (TIF) [file pone.0061256.s001.tif]

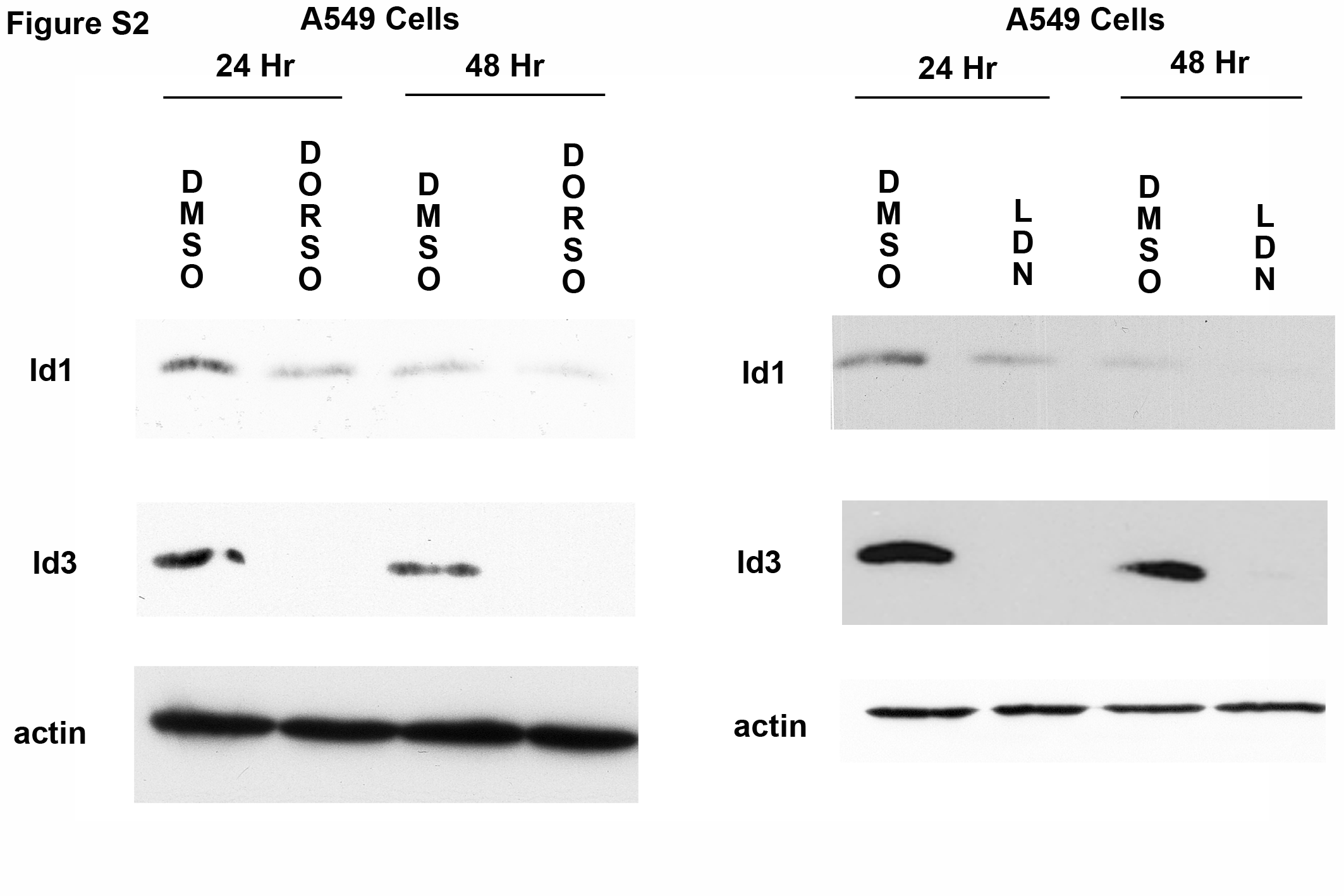

Supplement: Figure S2 — Dorsomorphin and LDN decrease protein expression of Id1 and Id3. Western blot analysis for Id1 and Id3 on A549 cells treated with 10 µM Dorsomorphin or 1 µM LDN for 24 and 48 hours. (TIF) [file pone.0061256.s002.tif]

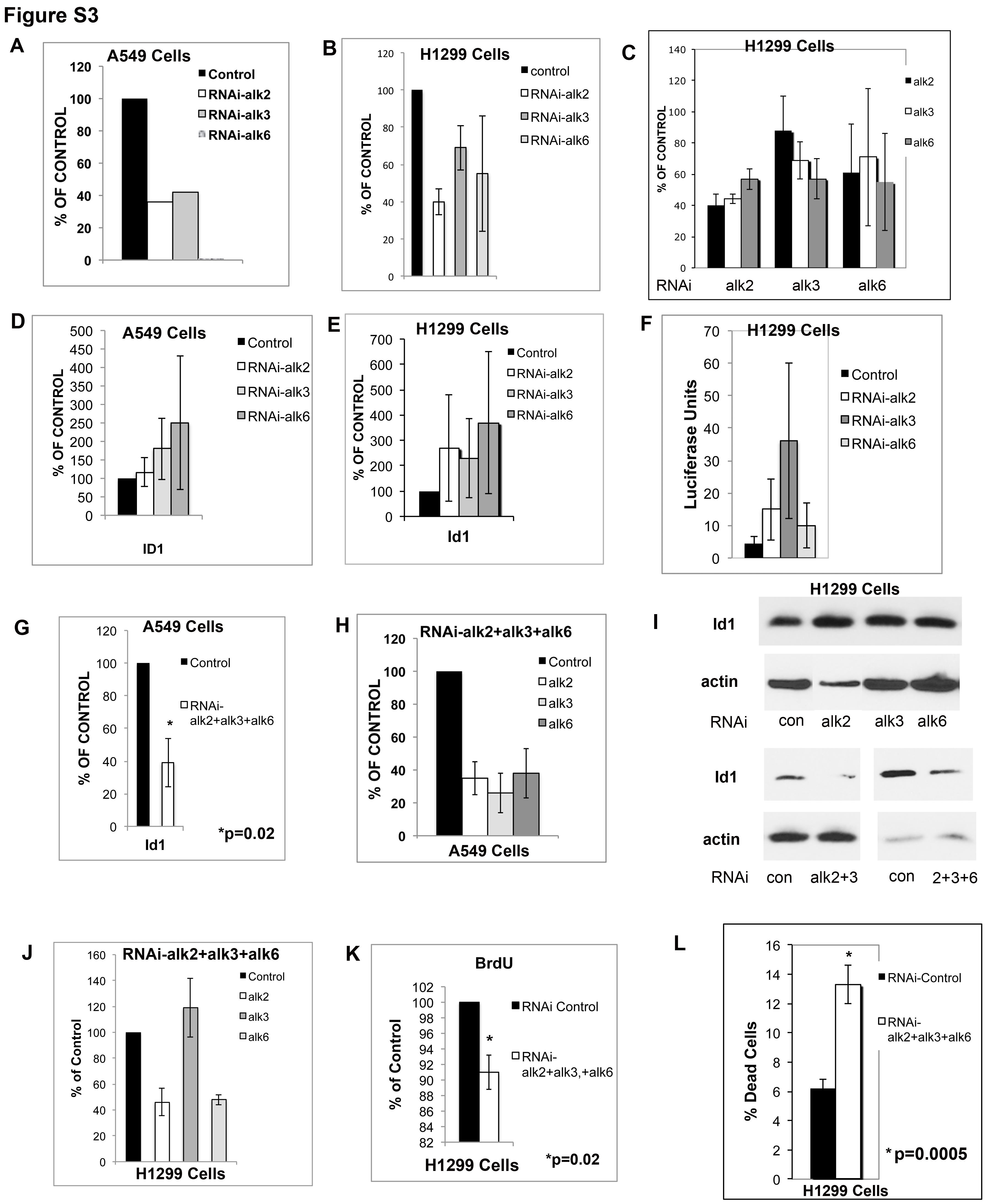

Supplement: Figure S3 — Knockdown of multiple BMP type I receptors using a second set of siRNA decreases BMP signaling, decreases proliferation, and induces cell death. (A–B) A549 and H1299 cells were transfected with siRNA targeting each type I BMP receptor and quantitative RT-PCR was performed for that BMP receptor. (C) Knockdown of each BMP type I receptor in H1299 cells was performed and quantitative RT-PCR performed for all 3 type I receptors. (D–E) A549 and H1299 cells were transfected with siRNA targeting a single type I BMP receptor or siRNA control. After 48 hours quantitative RT-PCR was performed for Id1. (F) H1299 cells were co-tranfected with BRE-luciferase reporter and siRNA for a single type I BMP receptor. After 48 hours luciferace activity was measured. (G) Knockdown of all type I BMP receptors was performed in A549 cells. Quantitative RT-PCR showed significant reduction in Id1 expression. (H) Quantitative RT-PCR shows a reduction of all 3 BMP type I receptors. (I) Western blot analysis for Id1 in H1299 cells with knockdown of a single type I BMP receptor or combination knockdown of alk2 and alk3, or all 3 BMP type I receptors. Studies show silencing more than one receptor is required to decrease Id1 expression. (J) Transfection of H1299 cells with siRNA targeting all type I receptors causes significant reduction of alk2 and alk6 with a corresponding significant reduction in (K) proliferation and (L) induction of cell death. (B,C,D,E,G,H,J,K) Data represents the mean of at least 3 experiments reported as the percent of control treated cells. (F,L) Data represents the mean of at least 3 experiments. (TIF) [file pone.0061256.s003.tif]

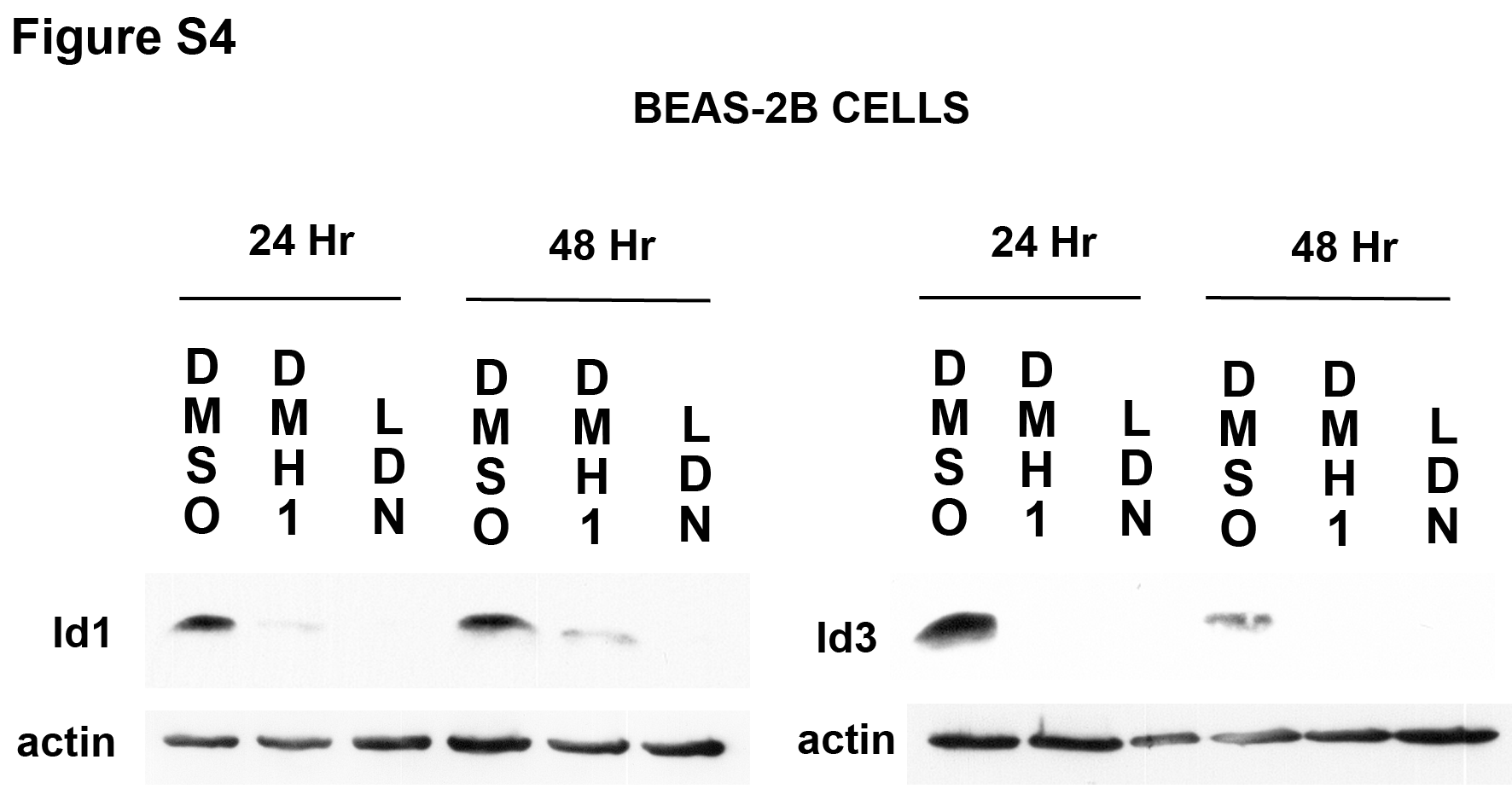

Supplement: Figure S4 — Western blot analysis showing immortalized normal human bronchial epithelial (BEAS-2B) cells treated with BMP receptor antagonists causes a significant reduction in the expression of Id1 and Id3. (TIF) [file pone.0061256.s004.tif]
